# Supplementary material for: Thalamo-accumbal circuit adaptations following extended oxycodone abstinence
Source: eLife. 2026 Aug 4;14:RP102189. doi: 10.7554/eLife.102189 (PMC13436959; doi:10.7554/eLife.102189)
Supplement: Supplementary file 2 [file elife-102189-repstand1.pdf]

NOTE: Please save this file locally before filling in the table, DO NOT work on the file within your internet browser as changes will not be saved. Adobe Acrobat Reader (available free here) is recommended for completion.

# The ARRIVE guidelines 2.0: author checklist

## The ARRIVE Essential 10

These items are the basic minimum to include in a manuscript. Without this information, readers and reviewers cannot assess the reliability of the findings.

| Item | | Recommendation | Section/line number, or reason for not reporting |
|---|---|---|---|
| **Study design** | 1 | For each experiment, provide brief details of study design including: | |
| | | a. The groups being compared, including control groups. If no control group has been used, the rationale should be stated. | See Methods: Subjects and Statistical Analysis; Results and figure legends throughout. |
| | | b. The experimental unit (e.g. a single animal, litter, or cage of animals). | See Methods: Ex vivo electrophysiology and optogenetic stimulation; Statistical Analysis. |
| **Sample size** | 2 | a. Specify the exact number of experimental units allocated to each group, and the total number in each experiment. Also indicate the total number of animals used. | See Results and figure legends throughout for exact n per group. |
| | | b. Explain how the sample size was decided. Provide details of any *a priori* sample size calculation, if done. | See Methods: Statistical Analysis; Results: intrinsic excitability section for post-hoc power analysis. |
| **Inclusion and exclusion criteria** | 3 | a. Describe any criteria used for including and excluding animals (or experimental units) during the experiment, and data points during the analysis. Specify if these criteria were established *a priori*. If no criteria were set, state this explicitly. | See Methods: Statistical Analysis; Morphological Analysis. Criteria were established a priori. |
| | | b. For each experimental group, report any animals, experimental units or data points not included in the analysis and explain why. If there were no exclusions, state so. | See Methods: Statistical Analysis. No unexpected exclusions occurred. |
| | | c. For each analysis, report the exact value of *n* in each experimental group. | See Results and figure legends for exact n per group per analysis. |
| **Randomisation** | 4 | a. State whether randomisation was used to allocate experimental units to control and treatment groups. If done, provide the method used to generate the randomisation sequence. | See Methods: Statistical Analysis. |
| | | b. Describe the strategy used to minimise potential confounders such as the order of treatments and measurements, or animal/cage location. If confounders were not controlled, state this explicitly. | See Methods: Behavioral methods; Ex vivo electrophysiology and optogenetic stimulation. |
| **Blinding** | 5 | Describe who was aware of the group allocation at the different stages of the experiment (during the allocation, the conduct of the experiment, the outcome assessment, and the data analysis). | See Methods: Behavioral methods; Statistical Analysis. |
| **Outcome measures** | 6 | a. Clearly define all outcome measures assessed (e.g. cell death, molecular markers, or behavioural changes). | See Methods: Behavioral methods; Ex vivo electrophysiology and optogenetic stimulation; Morphological Analysis. |
| | | b. For hypothesis-testing studies, specify the primary outcome measure, i.e. the outcome measure that was used to determine the sample size. | See Methods: Statistical Analysis; Results: intrinsic excitability section. |
| **Statistical methods** | 7 | a. Provide details of the statistical methods used for each analysis, including software used. | See Methods: Statistical Analysis. |
| | | b. Describe any methods used to assess whether the data met the assumptions of the statistical approach, and what was done if the assumptions were not met. | See Methods: Statistical Analysis. |
| **Experimental animals** | 8 | a. Provide species-appropriate details of the animals used, including species, strain and substrain, sex, age or developmental stage, and, if relevant, weight. | See Methods: Subjects. |
| | | b. Provide further relevant information on the provenance of animals, health/immune status, genetic modification status, genotype, and any previous procedures. | See Methods: Subjects; Surgeries. |
| **Experimental procedures** | 9 | For each experimental group, including controls, describe the procedures in enough detail to allow others to replicate them, including: | |
| | | a. What was done, how it was done and what was used. | See Methods: Surgeries; Behavioral methods; Ex vivo electrophysiology and optogenetic stimulation. |
| | | b. When and how often. | See Methods: Behavioral methods; Ex vivo electrophysiology and optogenetic stimulation. |
| | | c. Where (including detail of any acclimatisation periods). | See Methods: Subjects; Surgeries; Behavioral methods. |
| | | d. Why (provide rationale for procedures). | See Introduction and Methods throughout. |
| **Results** | 10 | For each experiment conducted, including independent replications, report: | |
| | | a. Summary/descriptive statistics for each experimental group, with a measure of variability where applicable (e.g. mean and SD, or median and range). | See Results and figure legends throughout. Data presented as mean ± SEM. |
| | | b. If applicable, the effect size with a confidence interval. | See Results: intrinsic excitability section. Cohen's d = 1.18 reported for male prolonged abstinence comparison. |
