# Supplementary material for: Thalamo-accumbal circuit adaptations following extended oxycodone abstinence
Source: eLife. 2026 Aug 4;14:RP102189. doi: 10.7554/eLife.102189 (PMC13436959; doi:10.7554/eLife.102189)
Supplement: Peer review history

# Editor report: eLife Assessment

**DOI:** 10.7554/eLife.102189.4.sa0

**Authors:** Natalie E Zlebnik

**Affiliations:** University of California, Riverside United States

**Keywords:** Compelling, Valuable

This **valuable** manuscript by Alonso-Caraballo et al is a novel piece of work that examines the impact of oxycodone self-administration on neural plasticity within paraventricular thalamic (PVT) to nucleus accumbens shell (Shell) pathway - two regions shown to play a key role in cue-induced drug seeking on their own - and whether this plasticity varies based on abstinence period and biological sex. Data show that a clinically relevant long-access model of self-administration promotes dependence in both male and female rats and provide **compelling** data that when compared to current literature indicate that craving-induced relapse for opioids may develop faster and may be more pronounced in females compared to males. In addition to these behavioral findings, the authors provide the first evidence that glutamate signaling within the PVT-to-Shell pathway is selectively strengthened at the output medium spiny neurons by opioids following protracted, but not acute abstinence. These data highlight a potential role for these adaptations in relapse behavior and identify a potential therapeutic target during abstinence to reduce relapse risk in abstaining individuals.

# Referee report: Reviewer #1 (Public review)

**DOI:** 10.7554/eLife.102189.4.sa1

[Editors' note: this version has been assessed by the Reviewing Editor without further input from the original reviewers. The authors have made minor revisions to address the comments raised in the previous round of review.]

Summary:

This manuscript by Alonso-Caraballo et al, is a novel piece of work that examines the impact of oxycodone self-administration on neural plasticity within paraventricular thalamic (PVT) to nucleus accumbens shell (Shell) pathway - two regions shown to play a key role in cue-induced drug seeking on their own, and whether this plasticity varies based on abstinence period and biological sex.

Strengths:

The authors show using a clinically relevant long-access model of opioid self-administration promotes dependence and acute withdrawal in both male and female rats. During subsequent cue-induced relapse tests at 1 or 14-days following the conclusion of self-administration, data show that while both male and females demonstrate drug-seeking behavior at both time points, females show a further elevation in responding on day 14 versus day 1 that is not observed in the males. When accounting for past work showing elevations in drug seeking in males after 30 days, these data indicate that craving-induced relapse for opioids may develop faster and may be more pronounced in females compared to males.

These behavioral findings were paralleled by use of ex vivo acute slice electrophysiology and circuit-specific ex vivo optogenetics to examine the impact of oxycodone self-administration on synaptic strength within the paraventricular thalamus (PVT) to nucleus accumbens shell (NAcSh) pathway(s). Data support a time-dependent but sex independent strengthening of glutamatergic signaling at PVT-to-NAcSh medium spiny neurons (MSNs) that is only present following a relapse test at 14 days post abstinence in males versus females, providing the first evidence that opioid self-administration and/or cue-induced drug-seeking augments this pathway. Using an extensive set of physiological measures, the authors show that this increased synaptic strength reflects a upregulation of presynaptic release probability. Further, this upregulation of excitatory signaling aligned temporally with an increase in MSN excitability, as assessed by increases in action potential firing frequency. Finally, the authors provide the first evidence that similar to other inputs to the NAcSh, PVT projections innervate both MSN as well as local interneurons, promoting a GABA-A specific feedforward inhibitory circuit. Interestingly, unlike direct excitatory inputs to MSNs, no changes were observed ostensibly within this feedforward circuit, highlighting a selective enhancement of excitatory drive and output of MSNs with protracted abstinence.

Overall, these data highlight a potential role for heightened synaptic strength within the PVT-NAcSh pathway in cue-induced relapse behavior during protracted abstinence and identify a potential therapeutic target during abstinence to reduce relapse risk in abstaining individuals.

Weaknesses:

Overall, the experimental approach and data provided appear rigorous and support their overall conclusions and achieve their goal of understanding how opioid self-administration impacts synaptic strength within the PVT-NAcSh pathway. Although not undermining these data, there are a few potential weaknesses that reduce the impact of the work. For example, the inability to directly assess whether cue-induced drug-seeking is in fact augmented compared to daily intake during self-administration in the maintenance face only permits the authors to denote that reexposure to cues and the context is sufficient to promote active lever pressing without demonstrating whether seeking behavior is in fact elevated further during a cue test. This is notably understandable as drug available sessions were 6-hours versus a 1hour relapse test. Importantly, it is clearly demonstrated that drug seeking is higher on average in female mice after 14 days versus 1 day.

With regard to interpretation of electrophysiology findings, the lack of inclusion of an abstinence only group does not permit interpretations to parse out whether observed increases in synaptic strength (or the lack of) reflect abstinence or an interaction between abstinence period and re-exposure to the operant chamber, as slices were taken 30-45 min post relapse test. While much literature has shown that drug induced adaptations in the NAc requires a post drug period for plasticity to measurably emerge, studies have also shown that re-exposure to heroin-associated cues following abstinence seemingly "reverses" increases in cell excitability in prelimbic-NAc pyramidal neurons (Kokane et al., 2023) and that depotentiation of morphine-induced increases in synaptic strength in the NAc shell can be depotentiated by drug re-exopsure -- an effect also observed with cocaine re-exposure (Madayag et al., 2019). Notably, the lack of effect at 14 but not 1 day supports the likelihood that the relapse test does not in fact influence the plasticity within the PVT-NAcSh circuit.

While the lack of effect on AMPAR:NMDAR ratio and rectification indices do support the notion that enhanced EPSC amplitudes in input-output curves do not reflect a change in AMPAR subunit expression (i.e., increased GluA2-lacking receptors that exhibit inward rectification at depolarized potential) nor a change in postsynaptic sensitivity to glutamate, without direct assessment of AMPAR-specific and NMDAR-specific input-output curves, it doesn't definitively exclude the possibility that both AMPA and NMDA receptor currents are being upregulated, thus negating an observable change in postsynaptic strength.

Overall, these findings provide novel insight into how the PVT-NAcSh pathway is altered by opioid self-administration and whether this is unique based on abstinence period and sex. Importantly, these were the primary objectives stated by the author. Data highlight a potential role for the observed adaptations in relapse behavior and identify a potential therapeutic target during abstinence to reduce relapse risk in abstaining individuals. However, it should be noted that no causal link is demonstrated without experiments to reduce/prevent relapse.

Comments on previous revisions:

The authors addressed previous concerns brought up, specifically by clarifying data interpretation as well as text modifications related to potential caveats of these interpretations.

# Referee report: Reviewer #2 (Public review)

**DOI:** 10.7554/eLife.102189.4.sa2

Summary:

This is an interesting paper from Alonso-Caraballo and colleagues that examines the influence of opioid use, acute and prolonged abstinence, and sex on cue-induced relapse and paraventricular thalamus (PVT) to nucleus accumbens shell (NAcSh) medium spiny neurons circuit physiology. The study presents a valuable finding that following prolonged, but not acute abstinence from oxycodone self-administration, female rodents exhibit higher relapse rates to drug paired cues. Additionally, the study presents the useful finding that prolonged abstinence increased PVT-NAcSh MSN synaptic strength in both sexes, an effect that is likely due to presynaptic adaptations. While the evidence to support these two findings is solid, further experiments are required to determine the functional role of the PVT-NAcSh MSN circuit in relapse following prolonged oxycodone abstinence, and the mechanism underlying the heightened relapse vulnerability in females in this model of opioid use disorder.

Strengths:

The paper is interesting, well written and presented, and the experiments are well designed and conducted. The revised analysis of spike count data that models the hierarchical structure of the data is appropriate to overcome low animal numbers and the potential for oversampling. The authors are transparent in reporting the results related to this analysis in figure 5 and acknowledge the study is underpowered to confirm the trend of increased intrinsic excitability in male MSNs following prolonged oxycodone analysis.

Impact:

The topic is of interest to the field of substance use disorders and gives solid evidence for the need to consider targeted therapeutics aimed at relapse prevention in opioid use disorder.

# Referee report: Reviewer #3 (Public review)

**DOI:** 10.7554/eLife.102189.4.sa3

Summary:

Alonso-Caraballo et al. use behavioral testing and ex vivo patch-clamp electrophysiology combined with circuit-specific optogenetic stimulation of PVT terminals to examine how oxycodone self-administration and abstinence duration shape cue-induced relapse and PVT-NAcSh synaptic transmission in male and female rats. In the revision, the authors reanalyzed intrinsic excitability using nested hierarchical GLMMs, acknowledged the low power in the male prolonged-abstinence group, and expanded the discussion of relevant PVT-NAc literature. These changes improve the manuscript. That said, most of the revisions are textual and the main experimental gap remains. Both sexes show increased oxycodone seeking compared to saline at 14 days, but only females show a time-dependent incubation from 1 to 14 days, and the PVT-NAcSh synaptic strengthening is the same in both sexes. Nothing in the revision brings those two observations closer together. The excitability data also come from NAcSh MSNs with no confirmation of PVT connectivity, which limits what circuit-specific conclusions can be drawn. The study is a solid characterization of abstinence-related synaptic changes in this pathway, but some of the conclusions still go further than the data allow.

Strengths:

The behavioral characterization is thorough and well-executed, covering self-administration, somatic withdrawal, and cue-induced relapse across two abstinence durations in both sexes. The sex-specific escalation in oxycodone seeking from 1 to 14 days in females but not males is a clear and compelling finding. The use of circuit-specific ex vivo optogenetics to isolate PVT terminal inputs onto NAcSh neurons is a genuine methodological strength, and the demonstration of feedforward inhibitory recruitment through local GABAergic interneurons adds meaningful novelty to the circuit characterization. The reanalysis of intrinsic excitability using nested hierarchical GLMMs appropriately accounts for the non-independence of cells recorded within the same animal and is a real improvement over the original approach. The expanded discussion of prior PVT-NAc work, particularly the more accurate treatment of Keyes et al. (2020) and Paniccia et al. (2024), better situates the findings within the existing literature.

# Author comment: Author response

**DOI:** 10.7554/eLife.102189.4.sa4

**Authors:** Yanaira Alonso Caraballo, Yan Li, Nicholas J Constantino, Megan A Neal, Gillian S Driscoll, Yunona Manasian, Grace K Cai, Maria Mavrikaki, Vadim Y Bolshakov, Elena Chartoff

**Affiliations:** University of Minnesota Minneapolis United States; McLean Hospital Belmont United States; Wake Forest School of Medicine North Carolina United States; McLean Hospital Belmont United States; Oregon Health and Science University Portland United States; McLean Hospital Belmont United States; McLean Hospital Belmont United States; Beth Israel Deaconess Medical Center Boston United States; Harvard Medical School Boston United States; Harvard Medical School, McLean Hospital Belmont United States

The following is the authors’ response to the previous reviews

**Reviewer #1:**

I recommend that the title be changed to not focus on sex differences to avoid misunderstanding.

We thank Reviewer #1 for this suggestion and agree that the original title could create a misleading impression. We have updated the title from "Sex-specific behavioral and thalamo-accumbal circuit adaptations after oxycodone abstinence" to "Thalamo-accumbal circuit adaptations following extended oxycodone abstinence" to more accurately reflect the scope of the findings.

The authors should also address the lack of difference physiologically compared to the behavior as a caveat more clearly in the discussion.

We thank the reviewer for this important suggestion. We have revised the Discussion to explicitly address this dissociation. Specifically, we added the following to the PVT-NAcSh synaptic strength section: " The absence of sex differences in PVT-NAcSh synaptic measures suggests that this pathway, as characterized here, represents a shared neuro-adaptation to prolonged oxycodone abstinence rather than a substrate for the heightened relapse vulnerability observed in females. The mechanisms driving sex-specific relapse likely involve additional circuit elements, such as sex hormone-dependent modulation, upstream inputs, or cell-type specific plasticity." This point is also summarized in the abstract.

**Reviewer #2:**

A major weakness of this study is the disconnect between the behavioral and neurophysiological data reported. While a striking sex difference in relapse-like behavior is observed, there are no statistically significant sex differences in any of the neurophysiological data reported. Moreover, without an experiment to functionally test the role of the PVT-NAc projection in relapse-like behavior following prolonged oxycodone, these two arms of the study seem divorced.

We respectfully disagree with the characterization that the behavioral and neurophysiological data are "divorced." The two arms of the study converge on a consistent and meaningful finding: PVT-NAcSh synaptic strength increases specifically after prolonged abstinence, this is the same time point at which enhanced cue-induced relapse is observed in both sexes. The absence of sex differences in synaptic measures does not weaken this convergence; it refines it by suggesting that circuit-level potentiation is a shared neuro-adaptation, while the sex-specific behavioral phenotype likely reflects additional modulatory mechanisms acting on this shared substrate. We have revised the Discussion to explicitly address this dissociation, as noted in our response to Reviewer #1 above. We acknowledge that functional manipulation of the PVT-NAcSh circuit would be required to establish causality, and we state this clearly in the manuscript.

In the introduction the authors state they aim to test the hypothesis that increased synaptic strength in PVTNAcSh projections are necessary for drug-seeking. This study does not include the required experiments to test this hypothesis.

We have revised the relevant section in the Introduction to accurately reflect the scope of our study: " We aimed to determine whether synaptic strength in PVT-NAcSh projections is affected following oxycodone abstinence and whether such changes are associated with cue-induced relapse and drug-seeking. Additionally, we examined whether there are sex-specific differences in either cue-induced relapse or PVT-NAcSh synaptic transmission after either 1 (acute) or 14 (prolonged) days of forced abstinence. Our results demonstrate that sex-specific enhancement in cue-induced relapse emerges after prolonged abstinence but not during acute abstinence from oxycodone self-administration. Although both males and females show increased cue-induced relapse after prolonged abstinence, females exhibited a greater relapse rate compared to males. Both sexes showed similar increases in PVT-NAcSh synaptic strength after prolonged abstinence, while synaptic strength was not altered after acute abstinence compared to saline controls. Together, these findings reveal a time-dependent increase in PVT-NAcSh synaptic strength and a sex-specific effect of prolonged abstinence on cue-induced relapse, while synaptic enhancements after prolonged abstinence were not sex-specific." This revision avoids implying a necessary or causal role for the circuit, which we did not test.

**Reviewer #3:**

The PVT-NAcSh synaptic strengthening after prolonged abstinence is statistically indistinguishable between sexes, while females but not males show a time-dependent escalation in oxycodone seeking from 1 to 14 days of abstinence. The Discussion proposes hormonal modulation or differences in upstream inputs as possible explanations, but none of these are tested and the gap is left unresolved.

We agree that the mechanistic basis of the behavioral sex difference remains an open question that the current study does not resolve. As noted in our response to Reviewer #1, we have revised the Discussion to explicitly acknowledge this dissociation and to clarify that PVT-NAcSh synaptic strengthening represents a shared neuro-adaptation rather than a mechanism specific to the female behavioral phenotype. We maintain that identifying this dissociation is itself a scientifically meaningful finding.

The intrinsic excitability recordings come from NAcSh MSNs with no confirmation that those neurons receive direct PVT input, which was raised in the original review, acknowledged in the revision, and not experimentally addressed.

We have added the following clarification to the excitability section of the Discussion: “It should be noted that the intrinsic excitability recordings were designed to characterize general properties of NAcSh MSNs following oxycodone abstinence, independent of their synaptic inputs. As such, the excitability data should be interpreted as reflecting changes in the NAcSh MSN population broadly rather than in PVT-connected neurons specifically. The standing theory suggests that MSN excitability decreases as a homeostatic response to increased glutamatergic input [23,43,58]. Our data do not support a compensatory decrease in excitability in either sex at either abstinence time point”. These recordings were never intended to be circuit-specific; the experiment was designed to characterize NAcSh MSN excitability at the population level, which is a valid and informative question in its own right.

The male prolonged-abstinence excitability trend has approximately 20% statistical power and is non-significant, yet the Discussion interprets it as a potential neuro-adaptation that could facilitate signal flow through the PVT-NAcSh circuit and contribute to relapse, which goes well beyond what the data support.

We have revised the relevant Discussion text to ensure the male excitability trend is interpreted appropriately. The revised text now reads: "In males, a non-significant trend toward increased excitability was observed after prolonged abstinence, with a large effect size (Cohen's d = 1.18); however, given that this group was substantially underpowered (approximately 20% power), this finding should be interpreted with caution and cannot be taken as evidence of a neuro-adaptation. Whether this trend, if confirmed in future studies with larger cohorts, reflects a broader MSN population response or is specific to PVT-connected neurons remains an open and interesting question." The speculative mechanistic interpretation previously present in this section has been removed.

The failure to distinguish between D1 and D2 MSNs remains a significant limitation given that cell-type specific plasticity at PVT-NAc synapses has been shown to be directly relevant to opioid seeking in prior work.

We agree that distinguishing between D1 and D2 MSNs would provide important mechanistic insight, and we acknowledge this explicitly as a limitation and a future direction in the Discussion. The use of transgenic Cre rat lines for cell-type-specific recording in a self-administration model requires significant additional infrastructure and was beyond the scope of the present study. This is precisely the direction our laboratory is currently pursuing, and the present findings provide empirical motivation for those experiments.

The Conclusion builds a mechanistic framework around D2 MSNs, PV interneurons, and D1 MSNs that is drawn from studies using different drugs or experimental designs, and none of these cell-type-specific mechanisms are tested in the present experiments.

We thank the reviewer for this important critique. We have revised the opening of the Conclusion to clarify that the cell-type-specific framework is grounded in prior literature and represents a hypothesis for future investigation rather than a conclusion drawn from the present data. The revised text now reads: " When considered alongside prior work, our findings highlight the need to examine the anatomical and cell-type specific organization of PVT inputs to the NAcSh in the context of opioid relapse. Based on existing literature, PVT projections onto D2 MSNs and PV interneurons may contribute to relapse vulnerability, while adaptations involving D1 MSNs may underlie incubation of craving, though these mechanisms remain to be directly tested in the oxycodone self-administration model used here". We believe this framing accurately represents the relationship between our findings and the broader literature without overstating what the present data demonstrates.
